# Utilization of ECMO with fiberoptic bronchoscopy for pediatric patients with lethal pulmonary hemorrhage unresponsive to conventional mechanical ventilation

**DOI:** 10.3389/fped.2025.1547579

**Published:** 2025-03-06

**Authors:** Siwei Lu, Yuelin Sun, Yingfu Chen, Yueqiang Fu, Jing Li, Chengjun Liu

**Affiliations:** Intensive Care Unit, Ministry of Education Key Laboratory of Child Development and Disorders, Chongqing Key Laboratory of Child Neurodevelopment and Cognitive Disorders, National Clinical Research Center for Child Health and Disorders, Children’s Hospital of Chongqing Medical University, Chongqing, China

**Keywords:** extracorporeal membrane oxygenation (ECMO), fiberoptic bronchoscopy, pulmonary hemorrhage, children, VA-ECMO, VV-ECMO

## Abstract

**Objective:**

To evaluate the safety and efficacy of extracorporeal membrane oxygenation (ECMO) combined with fiberoptic bronchoscopy in children with life-threatening pulmonary hemorrhage that does not respond to conventional mechanical ventilation.

**Methods:**

From October 2019 to June 2022, four pediatric patients with life-threatening pulmonary hemorrhage requiring ECMO support were admitted to our hospital. Based on their weight and vascular conditions, either venoarterial (VA)-ECMO or venovenous (VV)-ECMO was selected. The anticoagulation strategy was tailored, and fiberoptic bronchoscopy was performed to assess airway bleeding and remove blood clots.

**Results:**

The study involved four patients. Case 1 sustained injuries from a traffic accident, Case 2 experienced combined injuries from a high fall, Case 3 had pulmonary vascular malformation, and Case 4 presented with anti-neutrophil cytoplasmic antibody-associated vasculitis. Case 1 underwent VA-ECMO with carotid artery and vein cannulation, whereas the other patients received VV-ECMO with jugular–femoral vein cannulation. During cannulation, heparin was administered at 0.5 mg/kg. Protamine was subsequently used to neutralize heparin based on the bleeding situation. Anticoagulation was initiated 24 h after ECMO commencement in Cases 1, 2, and 4, maintaining an activated clotting time (ACT) of 160–180 s. In Case 3, active bleeding was observed in the tracheal tube post-ECMO initiation. Protamine was administered to reverse the effects of heparin, and anticoagulation was withheld for the first 72 h. After a second interventional embolization of the vascular malformations, the active bleeding ceased. Two fiberoptic bronchoscopies revealed no further bleeding, and anticoagulation was initiated at 5 U/kg/h to maintain an ACT of 160 s. Coagulation parameters, including ACT, blood analysis, and thromboelastography, were closely monitored, and heparin dosages were adjusted accordingly. Heparin was paused 1 h before each fiberoptic bronchoscopy and resumed afterward. During ECMO, all patients successfully underwent fiberoptic bronchoscopy. Cases 2 and 3 required three and six procedures, respectively. Substantial thrombi were removed from the airways of Cases 2 and 3. All patients survived, and they were discharged without complications related to ECMO or fiberoptic bronchoscopy.

**Conclusion:**

For children with life-threatening pulmonary hemorrhage that did not respond to conventional mechanical ventilation, the combination of ECMO and fiberoptic bronchoscopy represents a promising therapeutic option. ECMO rapidly corrects hypoxemia and provides respiratory support, whereas fiberoptic bronchoscopy effectively clears blood clots and facilitates lung re-expansion. Under an individualized anticoagulation strategy, this combined approach is both safe and effective, significantly improving clinical outcomes in pediatric patients with life-threatening pulmonary hemorrhage.

## 1 Introduction

Severe pulmonary hemorrhage resulting from thoracic trauma, pulmonary vascular anomalies, and anti-neutrophil cytoplasmic antibody (ANCA)-associated vasculitis is a life-threatening condition that can lead to acute hypoxemic respiratory failure. Despite the implementation of maximal medical interventions, including invasive mechanical ventilation, surgical drainage, and immunosuppressive therapy, acute progressive respiratory failure caused by this condition can result in fatal outcomes. Extracorporeal membrane oxygenation (ECMO), an advanced form of extracorporeal life support (ECLS), provides temporary cardiopulmonary function support for critically ill patients with refractory and reversible cardiac and/or pulmonary failure that does not respond to conventional therapies. During ECMO, systemic anticoagulation is essential to prevent thrombus formation and thromboembolism in the ECMO circuit. Consequently, the use of ECMO in patients with severe bleeding has traditionally been infrequent (1). However, advancements in ECMO technology have led to increasing reports demonstrating its efficacy in managing acute severe hypoxemia caused by pulmonary hemorrhage, including cases of multiple traumas, pulmonary vascular malformations, and diffuse alveolar hemorrhage (DAH) (2–4). Bronchoscopy, a safe procedure even during ECMO, plays a vital role in diagnosing and treating lung diseases by facilitating respiratory secretion clearance, determining the underlying pathology, and promoting lung re-expansion (5). In this study, we investigated the safety and efficacy of ECMO combined with fiberoptic bronchoscopy for the management of pediatric patients with severe pulmonary hemorrhage caused by chest trauma, pulmonary vascular malformations, and ANCA-associated vasculitis (AAV) treated at our institution since 2019. Our findings aim to provide insights to support clinical practice in managing such critical conditions.

## 2 Materials and methods

### 2.1 Study population

We enrolled four pediatric patients treated at our hospital between October 2019 and June 2022 for pulmonary hemorrhage resulting from trauma, pulmonary vascular malformation, or AAV. Table 1 presents the demographic characteristics of the four pediatric patients, including their sex, age, weight, and height. All patients presented with life-threatening severe hypoxemia that did not respond to conventional mechanical ventilation and met the criteria for ECMO support. This study was approved by our Institutional Review Board and Ethics Committee, and informed consent was obtained from all patients' parents or legal guardians.

**Table 1:** Demographic characteristics of patients undergoing ECMO.

Case	Gender	Age	Weight (kg)	Height (cm)
1	Male	2 years	11	90
2	Female	12.42 years	50	163
3	Male	8.83 years	25	135
4	Female	12.75 years	55	160

### 2.2 Intervention

Initial management involved endotracheal intubation, invasive mechanical ventilation, administration of antibiotics, and tailored supportive therapy to stabilize the patients.

The treatment and management of pulmonary hemorrhage adhered to established national and international guidelines, as well as expert consensus. Pre-ECMO interventions encompassed comprehensive airway management, respiratory and circulatory support, positional adjustments, fiberoptic bronchoscopy, and hemostatic therapies, including the administration of pituitrin and tranexamic acid. Blood products, such as red blood cell suspensions, fresh frozen plasma, cryoprecipitate, or platelets, were administered according to the patients' specific conditions. Fiberoptic bronchoscope-guided descending single-lung ventilation was attempted before ECMO initiation in Case 3. Post-ECMO management focused on minimizing sputum aspiration to prevent exacerbation of bleeding, continuing hemostatic treatment, withholding anticoagulation until hemostasis was achieved, and considering interventional therapies as needed.

### 2.3 Monitoring metrics

Monitoring included each child's heart rate (HR), invasive blood pressure, pulse oxygen saturation (SpO_2_), blood gas analysis, ECMO flow, speed, pump inlet pressure, membrane inlet and outlet pressures, pump inlet oxygen saturation, and hematocrit (HCT). Table 2 outlines the pre-ECMO blood gas and vital sign parameters, reflecting the most severe values observed in each case. Additional evaluations included chest x-ray and bedside ultrasound.

**Table 2:** Pre-ECMO initiation blood gas and vital sign parameters.

Case	PH ratio	HCO_3_^−^ (mmol/L)	PO_2_ (mmHg)	PCO_2_ (mmHg)	Lac mmol/L	HR (beats/min)	SBP (mmHg)	DBP (mmHg)
1	7.3	5.9	49	37	2.2	50	50	21
2	7.35	33.2	34	74	4.3	109	80	62
3	7.42	25.7	54.8	39.3	0.9	62	95	58
4	7.45	27.8	52.2	39.3	0.9	88	115	48

### 2.4 ECMO equipment and consumables

The ECMO setup utilized the Medos Deltastream MDC console with pediatric and adult kits and Medtronic ECMO arterial and venous cannulae. Specialized consumables included Medtronic monitors for blood SpO_2_ and HCT measurements.

### 2.5 Anticoagulation and monitoring

Heparin was used for anticoagulation. The anticoagulation monitoring variables included the activated clotting time (ACT), activated partial thromboplastin time (APTT), and complete blood count every 6 h. Table 3 summarizes the vasodilatory drugs administered prior to ECMO initiation. Thromboelastography (TEG) was performed as needed, and heparin dosages were adjusted according to the anticoagulation targets.

**Table 3:** Use of vasodilatory drugs before ECMO (µg/kg/min).

Case	Dobutamine	Epinephrine	Norepinephrine	Dopamine
1	15	0.1	-	-
2	-	0.05	0.9	10
3	-	-	-	-
4	-	-	-	5

“-” indicates that the drug was not used in the corresponding case.

### 2.6 Statistical analysis

Data analysis was conducted using SPSS version 25.0. Descriptive statistics were employed to summarize patient demographics, clinical characteristics, and outcomes. Continuous variables were expressed as the mean ± standard deviation or the median and interquartile range, depending on the data distribution. Categorical variables were summarized as frequencies and percentages. Given the small sample size (*n* = 4), no inferential statistical tests were applied, and the results were presented descriptively. The temporal trends in the partial pressure of arterial oxygen/fractional inspired oxygen (FiO_2_) ratio (P/F ratio), ACT, and mechanical ventilation duration were visualized using line charts for individual cases to highlight treatment effects and variability. Statistical significance was not assessed because of the limited sample size and the exploratory nature of the study.

## 3 Results

### 3.1 Participant characteristics

The study population included two boys and two girls aged 2–12.75 years. Their weight and height ranged 11–55 kg and 90–163 cm, respectively.

Case 1 was injured in a traffic accident, and he sustained predominantly thoracic injuries, including bilateral pneumothorax, lung compression, widespread pulmonary contusions, a small amount of left-sided effusion, and rib fractures on both sides (partial fractures on the left and multiple fractures on the right). Prior to the initiation of ECMO, bilateral closed thoracic drainage was performed. Despite elevated respiratory parameters and high-frequency ventilation after admission, the child's SpO_2_ continued to decline. Bright red blood was observed flowing from the tracheal tube, necessitating pressurized oxygen resuscitation bags to maintain chest movement. The child's HR and blood pressure remained extremely unstable.

Case 2 experienced injuries after a fall from the 11th floor of a building, including lacerations and bleeding in the liver, spleen, pancreas, and kidneys. The patient also sustained fractures at the inferior aspect of the C2 vertebral body, the left side of the arch of the C6 vertebra, the body of the T4 vertebra, and the right transverse processes of the T1–T4 vertebrae. Additionally, there were multiple fractures of both scapulae and a fracture at the anterior margin of the left acetabulum. These injuries were accompanied by bilateral pulmonary contusions, hemorrhage, and a 15% compression pneumothorax on the right side. The child underwent open exploratory laparotomy, partial resection of the right liver, splenectomy, and closed chest tube drainage on the right side at a local hospital.

Postoperative management included anti-infective therapy, hemostasis, and transfusion of blood products. Despite these measures, the child exhibited unstable vital signs, worsening pulmonary hemorrhage, and coagulation dysfunction on the day prior to ECMO initiation. This was accompanied by persistent severe hypoxia despite the use of high ventilator settings, elevated positive end-expiratory pressure (PEEP), and FiO_2_ of 100%.

Case 3 experienced pulmonary hemorrhage caused by a vascular malformation of the lungs. The patient initially presented with a persistent cough lasting 6 days and seven episodes of hemoptysis. Right pulmonary vascular malformation was suspected at another hospital, which recommended interventional treatment. Upon admission to our facility, the patient experienced severe coughing with massive hemoptysis within 20 min, accompanied by tachycardia, hypertension, decreased SpO_2_, cold extremities, profuse sweating, pale complexion, and cyanosis of the lips. Emergency endotracheal intubation and ventilatory support were initiated, and the patient was transferred to the pediatric intensive care unit (PICU). In the PICU, the child's pulmonary hemorrhage progressed rapidly, with continuous large volumes of fresh blood being evacuated through the endotracheal tube, resulting in hemorrhagic shock. Attempts at unilateral lung ventilation using a fiberoptic bronchoscope were unsuccessful, and severe hypoxia could not be effectively alleviated, even with high respiratory settings.

Case 4 experienced AAV-induced pulmonary hemorrhage. The patient presented with intermittent cough and fever lasting more than 3 weeks, which worsened in the 4 days before admission. These symptoms were accompanied by chest discomfort, pain, and dyspnea on the day before admission. CT performed at a local hospital revealed multifocal infectious lesions and partial lung consolidation bilaterally. Arterial blood gas analysis revealed a pH of 7.53, PCO_2_ of 32 mmHg, PO_2_ of 51 mmHg, and SpO_2_ of 90%. The hemoglobin concentration was 79 g/L, whereas the echocardiography findings were normal. Despite mechanical ventilation after admission, the patient's condition progressively declined, with persistent P/F ratios below 80 and an oxygenation index exceeding 25 for more than 10 h. Bronchoscopic lavage fluid analysis revealed iron-laden macrophages, and chest x-ray revealed diffuse ground-glass opacities and patchy consolidations across both lung fields. ANCA testing uncovered strong positivity for proteinase-3 (+++) with an antibody titer of 1:32 and an anti-proteinase-3 antibody level of approximately 382.4 U/ml, whereas myeloperoxidase antibodies and all other autoimmune markers were negative. The final diagnosis was AAV leading to pulmonary hemorrhage.

### 3.2 Initiation, operation, and mechanical ventilation of ECMO

The indications for ECMO in pulmonary hemorrhage were guided by the International ELSO Guidelines for pediatric respiratory and circulatory support, as well as the Chinese Expert Consensus on the application of extracorporeal membrane oxygenation in critically ill children. All patients met the criteria for ECMO support and respiratory assistance. The choice of the ECMO cannulation method was determined according to each patient's vascular condition.

Case 1 underwent veno-arterial (VA)-ECMO using the right internal jugular vein for cannulation. During the procedure, the patient experienced cardiac arrest, necessitating 10 min of cardiopulmonary resuscitation before ECMO was successfully initiated.

Cases 2–4 received veno-venous (VV)-ECMO via percutaneous cannulation of the right internal jugular and femoral veins. After successful cannulation, chest x-ray and echocardiography were performed to confirm the catheter position. Continuous monitoring of pre-pump, pre and post-membrane pressures was conducted to ensure proper function. Pre-pump pressure was maintained below −60 mmHg to secure adequate flow. ECMO equipment and the patients were regularly checked for mechanical and patient-related complications. Following ECMO initiation, the ventilation pressure and oxygen concentration were reduced for all patients, allowing their lungs to rest, the ventilator parameters for pediatric patients with pulmonary hemorrhage during ECMO were set in accordance with the International ELSO Guidelines and relevant literature. Adjustments were made according to the ECMO flow rate and the patients' blood gas parameters. Table 4 presents the mechanical ventilation settings and P/F ratios before and after ECMO initiation.

**Table 4:** Mechanical ventilation parameters and P/F ratio before and after ECMO initiation.

Case	Pre/Post-ECMO	Modes of mechanical ventilation	Frequency (breaths/min or Hz)	VT (ml)	PEEP (cm H_2_O)	FiO_2_	Platform pressure (cm H_2_O)	Amplitude	P/F ratio
1	Pre-ECMO	HOFV	8	–	–	100%	30	120	49
	Post-ECMO	VCV	28	60	10	35%	–	–	–
2	Pre-ECMO	VCV	18	400	16	100%	59	–	34
	Post-ECMO	VCV	16	180	10	50%	–	–	–
3	Pre-ECMO	VCV	20	240	10	100%	–	–	54.8
	Post-ECMO	VCV	20	120	10	50%	–	–	–
4	Pre-ECMO	VCV	20	400	14	100%	–	–	52.2
	Post-ECMO	VCV	15	250	10	45%	_	_	_

HOFV, high-frequency oscillatory ventilation; VCV, volume-controlled ventilation; “-” indicates unavailable or non-applicable data.

**Figure 1:**
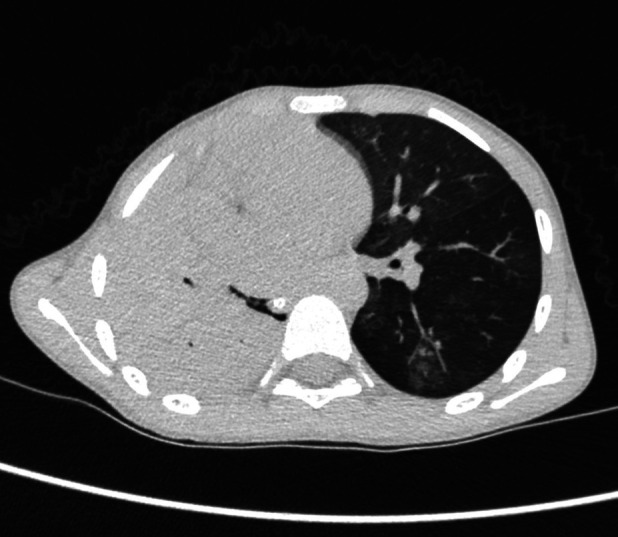
The initial CT scan at admission for case 3.

**Figure 2:**
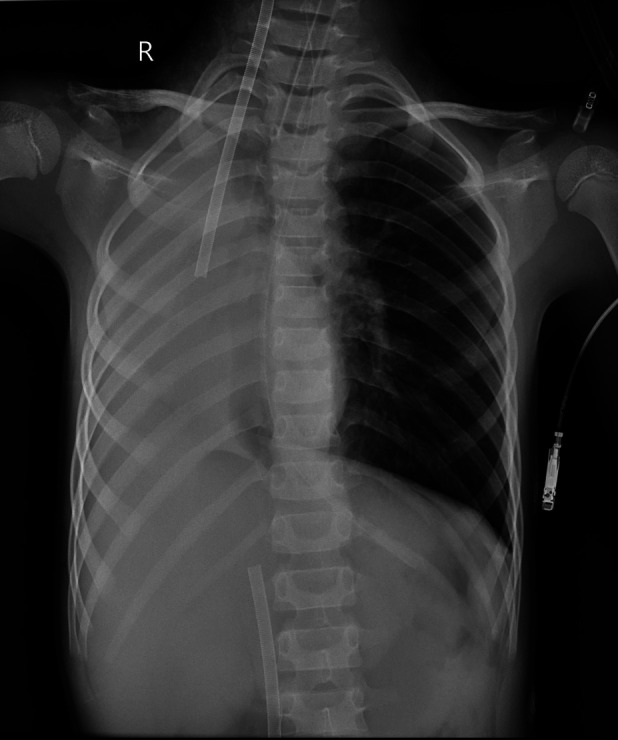
Chest x-ray performed immediately after ECMO initiation.

**Figure 3:**
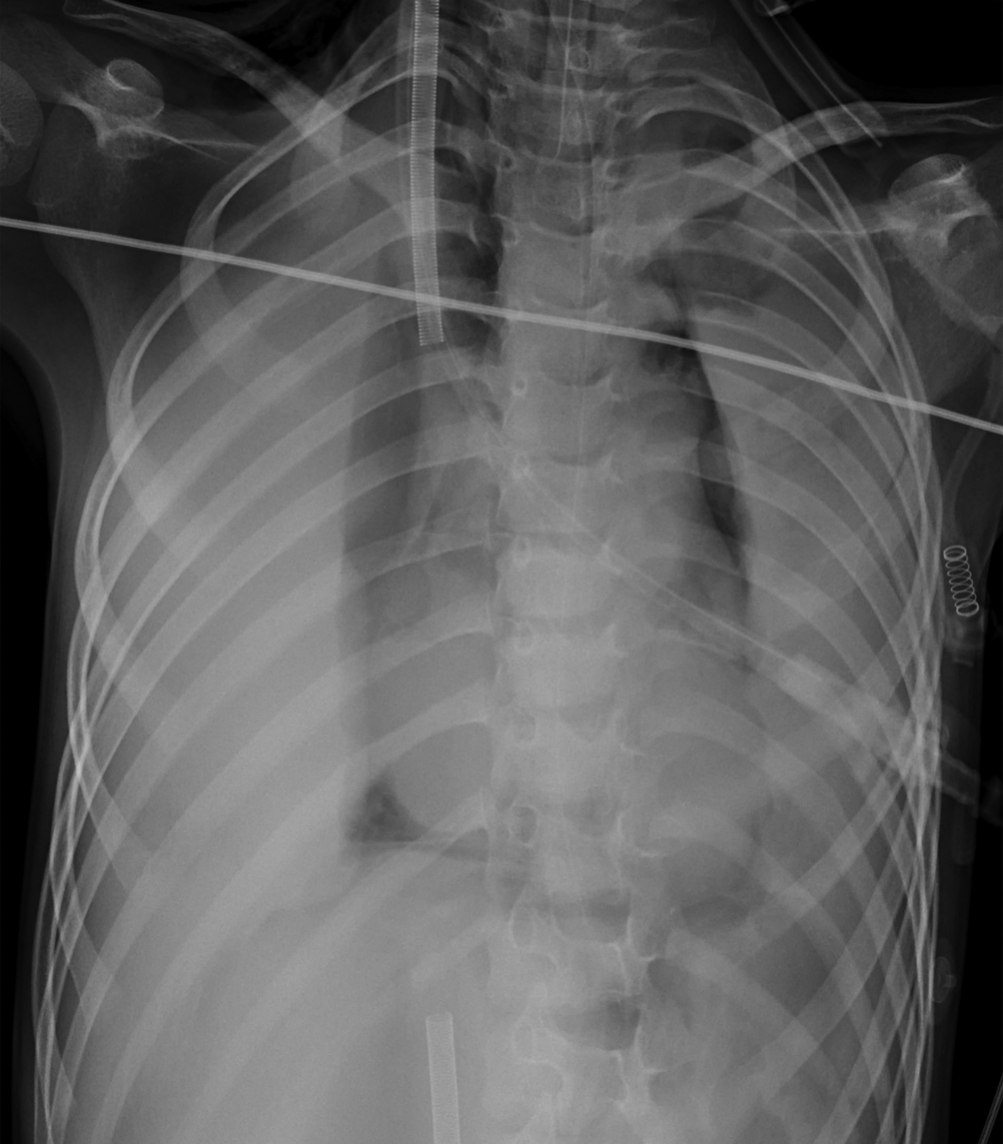
Case x-ray performed 42 h after ECMO initiation.

**Figure 4:**
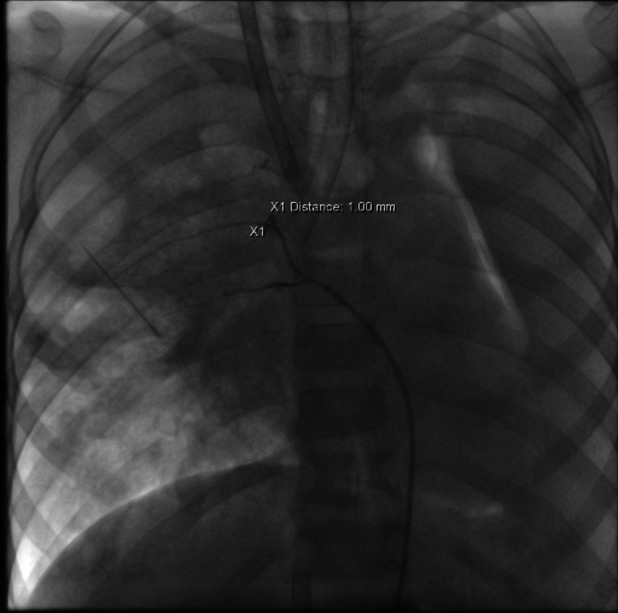
The abnormal vascular situation identified 48 h after ECMO initiation.

**Figure 5:**
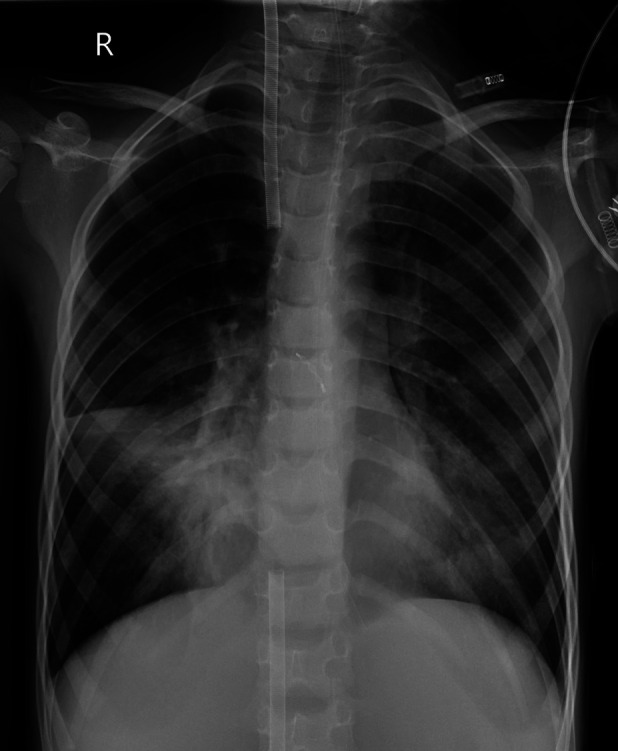
Chest x-ray performed 99 h after ECMO initiation.

**Figure 6:**
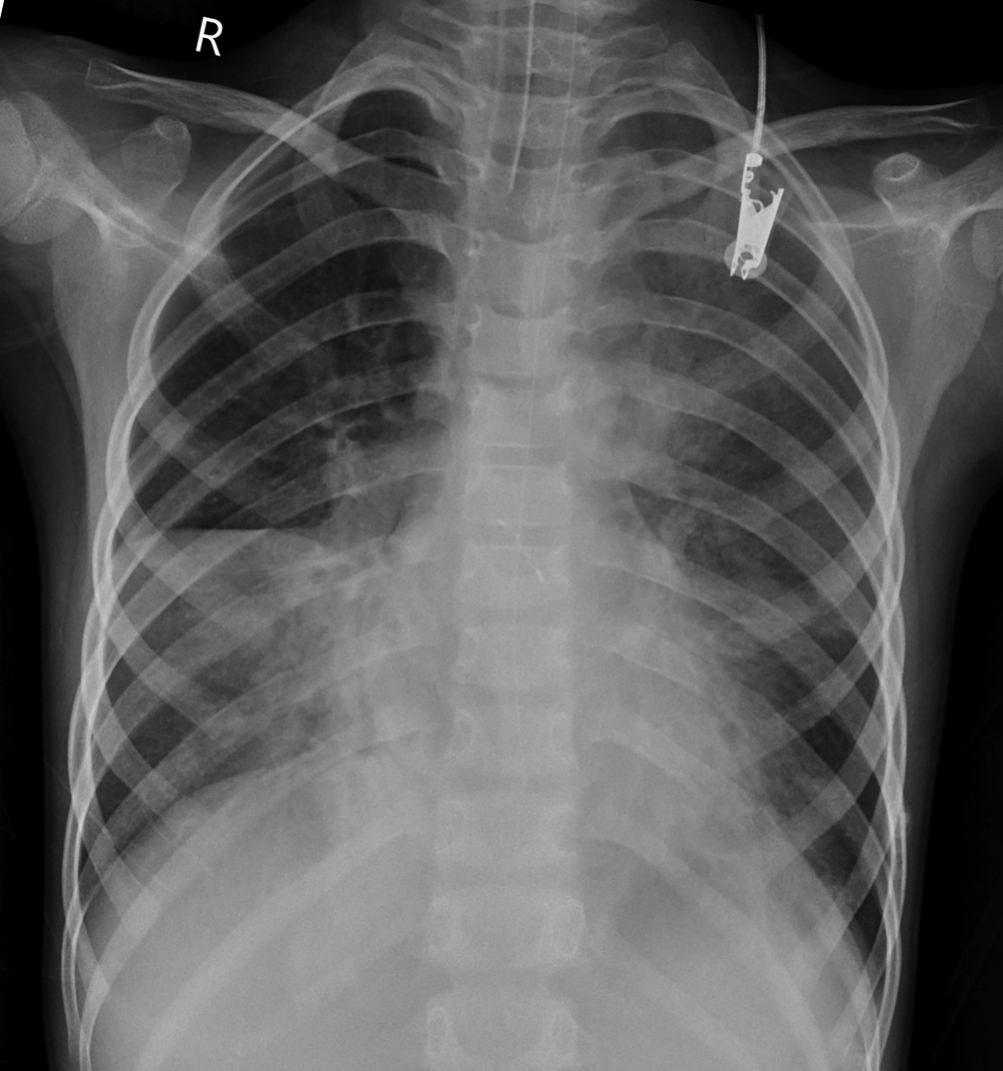
Chest x-ray performed 42 h after ECMO withdrawal.

Case 3 underwent an initial interventional examination 46 h after ECMO initiation because of worsening pulmonary hemorrhage. Figures 1–6 present the imaging findings for Case 3, including the CT and x-ray results before and after ECMO initiation, and angiographic details of vascular abnormalities. The examination identified a small collateral vessel approximately 0.8 mm in diameter originating from the fifth thoracic vertebral level of the ascending aorta and extending to the right pulmonary hilum. Additionally, an abnormal vessel measuring approximately 2.3 mm in width was observed at the right pulmonary hilum, along with a delicate, tortuous collateral vessel measuring approximately 1.0 mm in diameter that originated from the right subclavian artery and descended to the right pulmonary hilum through the right upper mediastinal space.

Additionally, angiography of the right and left pulmonary arteries revealed indistinct small dots and patches of contrast agent around the terminal branches of small arteries in each lobe of the right lung without evident abnormalities in the return of the right pulmonary veins. No significant abnormalities were observed in the pulmonary arteries and veins of the left lung. Fluoroscopy revealed diffuse reductions in lung transparency, mediastinal pneumothorax, and gas accumulation in the subcutaneous tissue of the right shoulder and neck, but the sealing attempt was unsuccessful.

ECMO was maintained for 54 h, but the bleeding from the abnormal vessels persisted, necessitating a second interventional procedure. During this procedure, a vessel measuring 1.1 mm in diameter was identified at the level of the eighth thoracic vertebra, and it branched into two intercostal arteries. The distal branches communicated with the pulmonary arteries at the hilum. The abnormal vessel was successfully occluded, as confirmed by postoperative angiography. After occlusion, no further bleeding was observed through the tracheal tube.

### 3.3 Regulation of anticoagulant therapy

A heparin anticoagulation protocol was implemented during ECMO management. Heparin was administered at a dose of 0.5 mg/kg during ECMO cannulation, with the need for protamine assessed according to each patient's bleeding status after ECMO initiation. For Cases 1, 2, and 4, heparin anticoagulation was not employed during the first 24 h post-ECMO initiation because of adequate blood flow. Continuous heparin anticoagulation was subsequently started to maintain an ACT of 160–180 s. In Case 3, active bleeding was observed in the tracheal tube following ECMO initiation. Protamine was administered to counteract the effects of heparin, and anticoagulation therapy was withheld during the first 72 h. After the second intervention successfully occluded the abnormal blood vessel, active bleeding ceased. Follow-up evaluations, including two fiberoptic bronchoscopies, revealed no evidence of ongoing bleeding. Anticoagulation was then initiated at a rate of 5 units/kg/h, maintaining an ACT of 160 s. Coagulation parameters, including ACT, complete blood counts, and TEG, were monitored regularly. Adjustments to the heparin infusion rate were made according to the monitoring results to ensure optimal anticoagulation management.

### 3.4 Fiberoptic bronchoscopy during ECMO

**Figure 7:**
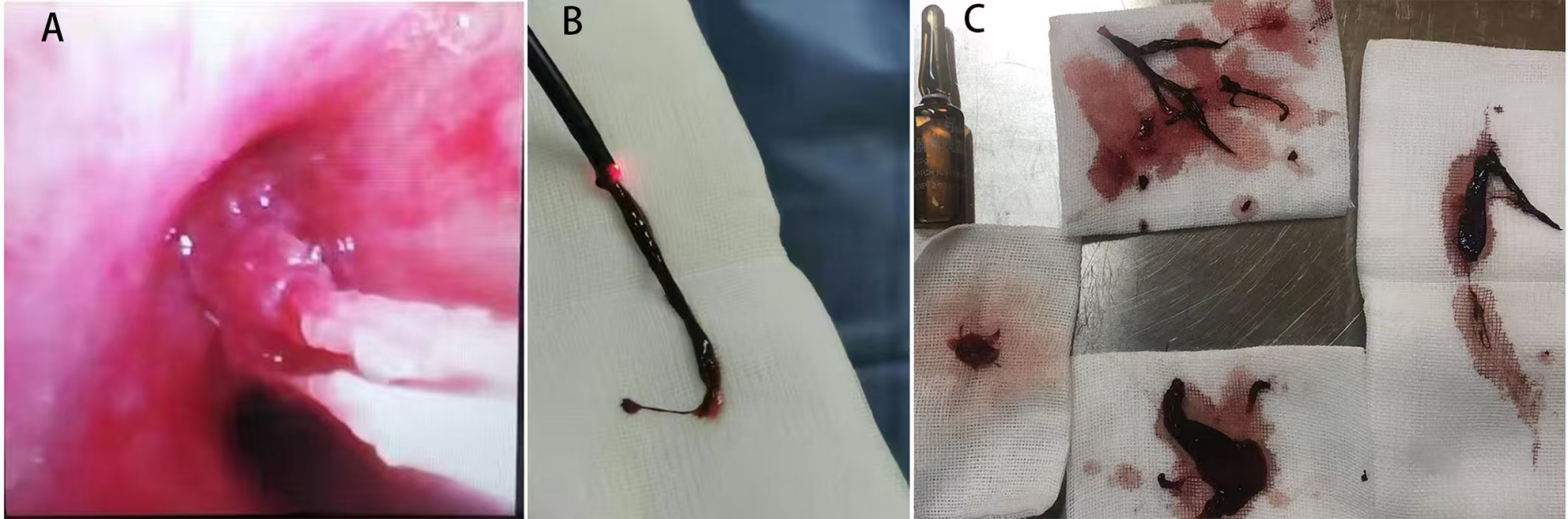
Images of blood clots extracted via fiberoptic bronchoscopy in Case 2. **(A)** Endobronchial thrombus observed during bronchoscopy. **(B)** Thrombus adherent to the distal tip of the fiberoptic bronchoscope during extraction. **(C)** Retrieved thrombus specimen.

**Figure 8:**
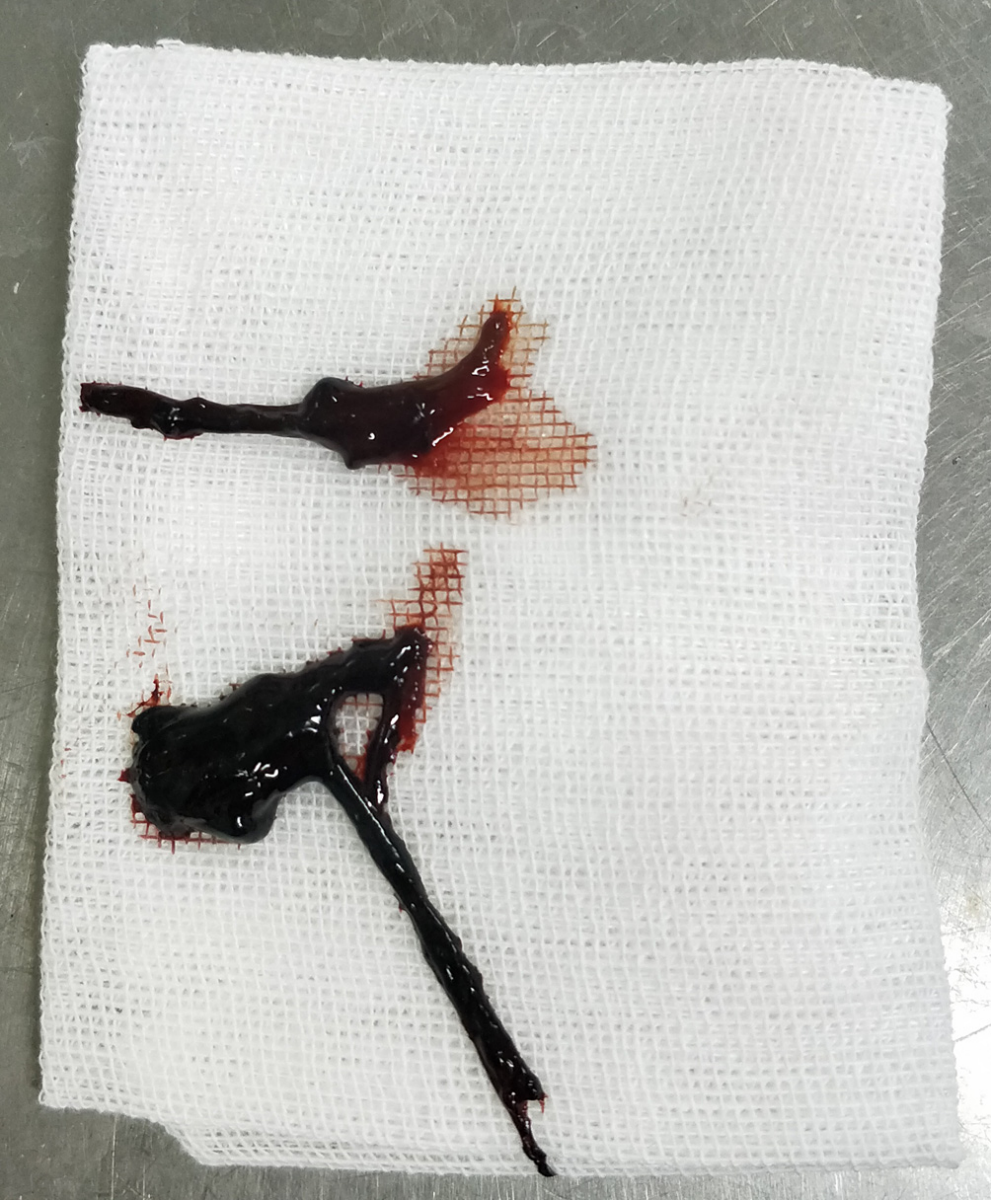
The blood clot extracted via fiberoptic bronchoscopy in case 3.

All four patients underwent fiberoptic bronchoscopy following the cessation of pulmonary hemorrhage during ECMO. Heparin infusion was discontinued 1 h prior to the procedure and resumed immediately afterward. No airway or pulmonary hemorrhage occurred during the bronchoscopic procedures in any patient. Case 1 underwent a single fiberoptic bronchoscopy, which revealed blood-tinged secretions covering the left upper lobe bronchi. Figures 7 and 8 depict the blood clots removed via fiberoptic bronchoscopy in Cases 2 and 3. Case 2 underwent three fiberoptic bronchoscopic examinations while on ECMO, which revealed significant aged blood clots in both the left and right mainstem bronchi and their branches. Large blood clots were removed using grasping and suctioning techniques, including one clot measuring up to 5 cm in length. Successive examinations revealed a gradual reduction in the clot burden. Following ECMO withdrawal, three additional bronchoscopic procedures were performed, ultimately achieving complete clearance of all residual thrombi and full lung re-expansion. Case 3 underwent six fiberoptic bronchoscopic procedures during ECMO. Thirty-four hours after ECMO initiation, an examination revealed continuous bright red blood flowing from the tracheal tube, obstructing visualization of the tracheal carina despite thorough suctioning. Two hours after the second intervention (56 h post-ECMO initiation), fiberoptic bronchoscopy revealed blood clots obstructing the central airway. These clots were removed using irrigation and biopsy forceps, allowing visualization of the tracheal carina and additional blood clots in the left bronchus. A tracheal tube was placed in the left main bronchus for unilateral lung ventilation. At 64 h (10 h after the second intervention), bronchoscopic examination disclosed active bleeding in the right bronchus and large blood clots in the left main bronchus. After suctioning, blood clots in the left lower lobe were cleared, although residual clots remained in the left upper lobe. Unilateral left-sided ventilation was continued after the procedure. Once heparin anticoagulation was initiated, three additional fiberoptic bronchoscopic examinations were performed, revealing the cessation of active bleeding in the right airway and the removal of residual clots. Following ECMO withdrawal, five more bronchoscopic examinations were conducted, confirming a gradual reduction in airway clots. Case 4 underwent one fiberoptic bronchoscopy that revealed a small amount of white mucous secretions in the airways.

### 3.5 Prognosis

No ECMO-related complications were observed during treatment, and the pulmonary hemorrhage did not worsen following ECMO initiation in Cases 1, 2, and 4. However, in Case 3, hemorrhage from the malformed vessels worsened after ECMO initiation, but it was successfully controlled following interventional embolization. Additionally, no exacerbation of pulmonary hemorrhage occurred during or after fiberoptic bronchoscopy. All patients were successfully weaned off both ECMO and mechanical ventilation, achieving full lung re-expansion. All patients survived and fully recovered, and they were discharged from the hospital in a completely recuperated state.

## 4 Discussion

With the advancement of society, the incidence of traffic accidents and falls resulting in injuries has risen. These high-energy injuries often involve multiple traumas characterized by complex and rapidly progressing conditions that frequently result in severe outcomes. They are commonly associated with hemorrhagic shock, respiratory and cardiac dysfunction, and, in some cases, progression to multiple-organ dysfunction syndrome. Studies reported that 50% of patients with closed trauma experience chest injuries (6, 7) involving life-threatening complications such as hemorrhagic shock and severe respiratory failure. When these injuries are accompanied by traumatic coagulopathy, the mortality rate can reach as high as 22% (8), whereas the mortality rate for traumatic acute respiratory distress syndrome (ARDS) ranges 10%–20% (9).

ECLS for trauma-induced ARDS was first reported in 1971 (10). However, in cases of closed trauma involving pulmonary contusion and concurrent organ injuries with bleeding, systemic anticoagulation poses a risk of exacerbating hemorrhage, making ECLS a potential contraindication (11). Larger-scale studies also revealed that ECMO is infrequently employed in patients with trauma. Nonetheless, hospital survival rates for patients with trauma who undergo ECMO range from 44% to 74.1% (12–15), comparable to the survival rate of 58% observed in adult patients requiring respiratory support (16).

With advancements in ECMO technology and the refinement of anticoagulation protocols, an increasing number of adults with severe trauma-induced cardiopulmonary failure are successfully undergoing ECMO both domestically and internationally, achieving favorable outcomes (2, 15). However, the application of ECMO in pediatric patients with severe trauma remains relatively limited (17, 18).

Hemoptysis is a common clinical symptom, with massive hemoptysis accounting for approximately 5% of all cases. This condition is critical and life-threatening, with a reported mortality rate within the respiratory system ranging from 6.5% to 38% (19). In children, massive hemoptysis is rare (20), with pulmonary vascular anomalies being one of the underlying causes. Without timely treatment, the mortality rate exceeds 50%. However, interventional and surgical procedures have been demonstrated to significantly reduce the mortality rate to 7%–18% (21).

The primary cause of death in children with massive hemoptysis is airway obstruction by blood and clots, leading to asphyxiation, sudden cardiac arrest, and respiratory and circulatory failure (22). Traditionally, massive hemoptysis was considered a contraindication for ECMO therapy. However, a study demonstrated that ECMO in children with pulmonary hemorrhage does not increase the incidence of bleeding and that it can even reduce bleeding within 24 h of initiation (23). Further research has indicated that under ECMO support, embolization or surgical procedures can be safely performed to treat pulmonary vascular malformations or vascular injury-induced pulmonary hemorrhage (24–26).

DAH is a rare but life-threatening condition, with approximately 10%–30% of patients with AAV at risk of developing DAH. It is the primary cause of hypoxic respiratory failure in patients with AAV (27). DAH is a significant complication of AAV and a strong predictor of early mortality, with reported 1-year mortality rates ranging from 18% to 50% (28). Mortality rates are even higher among patients requiring tracheal intubation and mechanical ventilation, reaching up to 77% (29). In cases of ANCA-related pulmonary hemorrhage with P/F ratios lower than 100, mortality rates exceed 80% (30).

Historically, bleeding was considered a relative contraindication for ECMO. However, recent studies have demonstrated that ECMO can be life-saving in patients with pulmonary hemorrhage caused by AAV-induced respiratory failure (28, 30–32).

Bronchoscopy, widely regarded as safe for patients on anticoagulant therapy, plays a pivotal role in diagnosing and managing lung diseases. During bronchoscopy in ECMO-supported patients, secure immobilization of the head and neck is essential to ensure proper positioning of the ECMO cannula (5, 33).

During ECMO, it is critical to remain vigilant for the potential risks of bleeding and thrombosis and to optimize anticoagulation strategies to ensure safe and effective treatment. Table 5 provides an overview of ECMO parameters, mechanical ventilation durations, and hospitalization periods, highlighting the efficacy of ECMO in managing severe pulmonary hemorrhage. Studies have demonstrated that ECMO can be temporarily performed without systemic anticoagulation (34), with this approach maintained for up to 36 h without heparin administration (35). Once hemorrhaging is controlled, anticoagulation should be initiated to prevent thrombosis within the ECMO circuit and oxygenator.

**Table 5:** ECMO parameters, mechanical ventilation, and hospitalization data.

Case	Drainage cannula (F)	Perfusion cannula (F)	Flow rate (L/min)	ECMO duration (h)	Mechanical ventilation before ECMO (h)	Total mechanical ventilation (h)	ICU stay (days)	Total hospitalization (days)
1	14	12	0.9	40.55	11.7	167.17	14	41
2	19	17	2.8	97.17	62.83	248.58	28	28
3	17	15	1.5	108.83	48.1	249.63	16	22
4	19	15	3	181.33	55.17	285.00	14	35

F, French gauge (size of cannula); Flow (L/min): The flow rate maintained during ECMO support. ICU and total hospitalization durations are presented in days for clarity.

The recommended target range for ACT is 160–180 s, whereas APTT should be maintained at 50–70 s. When these parameters are achieved, invasive investigations and surgical procedures can be performed with relative safety (36).

The study patients presented with pulmonary hemorrhage caused by chest trauma, vascular anomalies, and AAV, resulting in severe hypoxemia that was unresponsive to conventional treatments and that carried a high risk of mortality. Although pulmonary hemorrhage was previously considered a contraindication for ECMO, all patients successfully underwent ECMO therapy following the optimization of anticoagulation strategies.

In the initial phase of ECMO, systemic heparin anticoagulation was withheld for 24 h after ECMO initiation in Cases 1, 2 and 4 and for 72 h in Case 3 in the absence of bleeding symptoms. ACT was maintained at 160–180 s, with no bleeding or thrombosis-related complications observed during the treatment period.

All patients underwent fiberoptic bronchoscopy while receiving ECMO support. Cases 2 and 3 required multiple bronchoscopic procedures for blood clot removal. Additionally, Case 3 underwent anomalous vascular occlusion combined with unilateral lung ventilation under bronchoscopic guidance. These interventions were performed without any perioperative or postoperative hemorrhagic complications, demonstrating the safety and efficacy of this approach, consistent with findings in the existing literature.

In conclusion, ECMO provides adequate oxygen delivery, and fiberoptic bronchoscopy effectively removes airway blood clots and alleviates obstructions, thereby promoting lung re-expansion. For pediatric patients with life-threatening pulmonary hemorrhage, optimizing the anticoagulation strategy allows the combination of ECMO and fiberoptic bronchoscopy to serve as a safe and innovative therapeutic approach, offering improved outcomes in the management of this critical condition.

## Data Availability

The raw data supporting the conclusions of this article will be made available by the authors, without undue reservation.
